# Therapeutic Use of Mesenchymal Stromal Cells: The Need for Inclusive Characterization Guidelines to Accommodate All Tissue Sources and Species

**DOI:** 10.3389/fcell.2021.632717

**Published:** 2021-02-16

**Authors:** Adrienne Wright, Marne L. Arthaud-Day, Mark L. Weiss

**Affiliations:** ^1^Department of Anatomy and Physiology, Kansas State University, Manhattan, KS, United States; ^2^Department of Management, Kansas State University, Manhattan, KS, United States; ^3^Midwest Institute of Comparative Stem Cell Biotechnology, Kansas State University, Manhattan, KS, United States

**Keywords:** MSC, clinical translation challenge, metrology and characterization, commercialization, biotherapeutic development

## Abstract

Following their discovery over 50 years ago, mesenchymal stromal cells (MSCs) have become one of the most studied cellular therapeutic products by both academia and industry due to their regenerative potential and immunomodulatory properties. The promise of MSCs as a therapeutic modality has been demonstrated by preclinical data yet has not translated to consistent, successful clinical trial results in humans. Despite the disparities across the field, MSC shareholders are unified under one common goal—to use MSCs as a therapeutic modality to improve the quality of life for those suffering from a malady in which the standard of care is suboptimal or no longer effective. Currently, there is no Food and Drug Administration (FDA)-approved MSC therapy on the market in the United States although several MSC products have been granted regulatory approval in other countries. In this review, we intend to identify hurdles that are impeding therapeutic progress and discuss strategies that may aid in accomplishing this universal goal of widespread therapeutic use.

## Introduction

Multipotent mesenchymal stromal cells (MSCs) are a heterogeneous population that when expanded *in vitro* includes stem, progenitor, and differentiated cells. MSCs have been implicated as a therapeutic modality in tissue injuries, chronic degenerative disorders, and inflammatory diseases on account of their regenerative potential and anti-inflammatory properties (Friedenstein et al., 1968, 1970; Galipeau and Senséb, 2018). Although therapeutic use in humans is the end goal, preclinical research relies on animal models for proof of concept and technique development, and thus animal applications cannot be overlooked. The first isolation and culture of MSCs were performed using bone marrow from guinea pigs (the 1970s) and then extended to rats in the 1980s (Friedenstein et al., 1987; Owen and Friedenstein, 1988). Isolation and culture of human MSCs did not begin until the early 1990s (Haynesworth et al., 1992; Lazarus et al., 1995; Pittenger et al., 1999). Since then, MSCs have become a widely studied experimental therapeutic product tested in over 1300 registered clinical trials (clinicaltrials.gov “mesenchymal” 6/5/20) (Galipeau and Senséb, 2018). In human clinical trials, allogeneic MSCs have been consistently shown to be safe but have not been able to replicate the large effect sizes predicted from preclinical research. For this reason, small and large trials have failed to meet efficacy endpoints (Li et al., 2016; Galipeau and Senséb, 2018).

A vast preclinical dataset, from both *in vitro and in vivo* animal studies, supports the notion that MSCs are a potent cellular therapeutic agent. Here, we will review the *in vitro* preclinical data, but reviews of the *in vivo* preclinical data can be found here (Vu et al., 2014; Squillaro et al., 2016; Lukomska et al., 2019; Dave et al., 2020). Why is there such a gap between the expectations set by preclinical data and human MSC trials? The inconsistent results could be due to product irregularities, transferability across species, or poor estimation of effect size from preclinical data leading to insignificant findings. Our thesis here is that to move forward strategically, the MSC field needs to recognize and address shortcomings that have been given little consideration in the rush toward clinical development. Preclinical data needs to be strengthened in regards to its ability to be translated. Instead of continuing to produce inconsistent preclinical *in vitro* and *in vivo* data that poorly translates, effort should be placed on determining the root of the transferability issues so that consistent, reliable data can be generated allowing for replication across research laboratories. In addition, although the potential of MSCs remains undisputed, questions remain concerning the mechanisms-of-action (MOAs), how *in vitro* testing correlates to *in vivo* activity, the number of cells in a dose, the route of administration, and how all of this relates to the therapeutic effects for the various indications (Mendicino et al., 2014).

To do this, we believe that first, characterization guidelines need to be updated to accommodate different MSC populations. This includes addressing variations in the literature that may obscure rather than explain MSC's physiological effects that impact therapeutic response. These inconsistencies include, but are not limited to, MSC tissue source and species-to-species differences. Second, along with updated characterization guidelines, improved standardization in the field would help to eliminate product and lot-to-lot variation as well as address the concern of purity vs. potency. Lastly, to properly address these concerns, more research funding is required. With federal funding on research and development (R&D) declining, and businesses spending over three times the amount of the federal government on R&D, it is clear that industry-sponsored research is critical. Businesses are more prone to fund research that has commercial applicability rather than research that simply addresses a question (Sargent, 2020). By focusing research efforts on areas with commercial potential, not only could this increase research funding but also could decrease time to market.

## Challenges for Clinical Translation of Mesenchymal Stromal Cells

### Outdated Characterization Guidelines

In the early 1990s, Arnold Caplan was the first to use the term “mesenchymal stem cell” to describe the cells involved in embryonic bone and cartilage formation as well as repair and maintenance in the adult (Caplan, 1991). Following this discovery, many researchers argued that there was no feasible way to prove whether the *in vitro* cultured MSCs contained stem cells and, because of this, suggested alternative terms to label these cells. Although we still see the term “mesenchymal stem cells” used in literature more than 25 years later, the ISCT released a position piece in 2005 stating that the proper designation for these cells should be a multipotent mesenchymal stromal cell, seeing as they are a heterogeneous population in which not all cells have stem-like properties (Horwitz et al., 2005).

Following the nomenclature article, the ISCT's MSC working group released “minimal criteria” that should be demonstrated before a cell can be considered or referred to as an MSC (Dominici et al., 2006). These simplified guidelines include (1) Tissue culture plastic adherent; (2) Positive (≥95%) for surface antigen markers CD105, CD90, and CD73 while also negative (≤2%) for CD45 (pan-leukocyte), CD34 (hematopoietic and endothelial cells), CD14 or CD11b (monocytes and macrophages), CD79α or CD19 (B cells), and HLA-DR; and (3) Capable of differentiation to adipocytes, chondroblasts, and osteoblasts (Dominici et al., 2006). This definition is 14 years old and yet still widely used today. Although many researchers do go beyond this minimal definition, many also DO NOT meet this minimum.

The lack of uniformity has contributed to inconsistencies within the field. As noted by Mendicino et al., the current MSC guidelines used for characterization are not distinctive and therefore may not adequately define the cells and their biological function (Mendicino et al., 2014). Furthermore, this simplified definition does not consider species differences, tissue source, and passage of cells at the time of characterization, pointing to the need for refinement or updating of the “minimal criteria.” In 2013, the ISCT amended the MSC definition to include a bioassay of immunosuppressive properties, but it did not refine the original definition. In 2019, ISCT updated their MSC definition to suggest (1) including the tissue origin of cells, (2) use of stromal cell nomenclature unless rigorous evidence for stemness is shown, and (3) including functional assays to define therapeutic mechanism of action, but no tissue-specific guidelines were addressed (Viswanathan et al., 2019). Although the ISCT suggestions exist, there has been no enforcement of the issue by academic journals. We suggest that the ISCT follow the International Society for Extracellular Vesicles (ISEV) and the Functional Genomics Data Society (FGED) and establish their own unique set of minimally accepted publication criteria (Brazma et al., 2001; Théry et al., 2018).

### Biological Variability Translates to MSC Inconsistencies

To simply focus research on commercial use is only part of the picture. Science, either basic science or translational research, depends upon the ability to replicate published work, and hopefully, to extend that work. This includes observational research and hypothesis-driven research. As such, science depends upon the control of experimental variables, and minimizing experimental error. One issue in biology is that certain variables are inherently “variable” due to the complexity of the system, and this adds intricacy to the metrology (the science of measurement).

Historically, problems associated with cell culture have had a significant impact on the field of biology. Issues such as misidentification, the use of contaminated cell cultures (e.g., mycoplasmas), or the effects of phenotypic drift have led to the creation of guidelines that not only highlight the problems, but also provide guidance on how to avoid or eliminate the issues. In some countries, legislation or codes of practice govern research since it interacts with both ethical and scientific boundaries. For example, in stem cell research, the production of new human embryonic cell lines was restricted in the US, forcing science institutions, many which were federally-funded, to use only existing embryonic lines. The result of these sanctions was that researchers were only able to use a handful of preexisting lines that were easy to propagate and make available, thus forcing standardization of the industry. Although this means of standardization was extreme, it still allowed the field to conform thereby inducing reproducible research. Although standardization is not required by the FDA for clinical use, MSC stakeholders should support standardization efforts as it would benefit the field by allowing for more meaningful comparisons among studies, thus allowing for a smoother clinical translation (Mendicino et al., 2014). Further, replication as a result of standardization would allow for more efficient research, consequently transferring to cost savings.

### Regulatory Gaps in MSC Therapy

Currently, there are ten approved MSC therapies worldwide (Table 1) on the market for various indications, yet not a single FDA-approved product for use in the United States (Pereira Chilima et al., 2018; Levy et al., 2020; Shammaa et al., 2020). Differences in regulatory approvals around the globe have left gaps where some countries have approved products that have been on the market for over 10 years and other countries still have yet to grant approval to an MSC product. All countries with approved MSC products have a governing body, similar to the FDA, that has regulatory oversight of cell therapy products. Although similar, each country governs their own unique set of regulations and approval processes. These processes are reviewed in depth here (Ancans, 2012; Choi et al., 2015; Ridgway et al., 2015; Nagai and Ozawa, 2017; Tiwari and Desai, 2018; Mendicino et al., 2019; O'Sullivan et al., 2019). To alleviate gaps, some have suggested that the World Health Organization (WHO), an agency within the United Nations (UN), is a logical choice to develop guidelines and recommendations for the Member States (Petricciani et al., 2017). Although not a regulatory authority, WHO has a mandate to advance and advocate for international standards involving biological and pharmaceutical products, and many countries look to WHO for guidance in developing guidelines (Petricciani et al., 2017).

**Table 1:** MSC products with regulatory approval (Pereira Chilima et al., 2018; Levy et al., 2020).

**MSC product (company)**	**Approval granted (year)**	**Indication**	**Product type**
Queencell (Anterogen Co. Ltd.)	South Korea (2010)	Subcutaneous tissue defects	Autologous human AT-MSC
Cellgram-AMI (Pharmicell Co. Ltd.)	South Korea (2011)	Acute myocardial infarction	Autologous human BM-MSC
Cartistem (Medipost Co. Ltd.)	South Korea (2012)	Knee articular cartilage defects	Allogeneic human UC-MSC
Cupistem (Anterogen Co. Ltd.)	South Korea (2012)	Crohn's fistula	Autologous human BM-MSC
Prochymal, remestemcel-L (Osiris Therapeutics Inc., Mesoblast Ltd.)	Canada (2012)	GvHD	Allogeneic human BM-MSC
	New Zealand (2012)		
Neuronata-R (Corestem Inc.)	South Korea (2014)	Amyotrophic lateral sclerosis	Autologous human BM-MSC
Temcell HS (JCR Pharmaceuticals)	Japan (2015)	GvHD	Allogeneic human BM-MSC
Stempeucel (Stempeutics Research PVT)	India (2016)	Critical limb ischemia	Allogeneic human BM-MSC
Alofisel (TiGenix NV/Takeda)	Europe (2018)	Complex perianal fistulas in Crohn's disease	Allogeneic human AT-MSC
Stemirac (Nipro Corp)	Japan (2018)	Spinal cord injury	Autologous human BM-MSC

In the US, culture-expanded MSC-like cells are considered to be a more-than-minimally-manipulated cellular and gene therapy (CGT) product regulated by section 351 of the Public Health Service (PHS) Act 42 U.S.C.262 (Galipeau et al., 2016). Due to this designation, MSC-like cells require an Investigational New Drug (IND) application and approval from the FDA to be used in a clinical trial (Galipeau et al., 2016). Under this regulation, a test to measure potency as part of the release criteria is required although standardization among the field and ISCT minimal criteria are not required (Food and Drug Administration, 2011b; Galipeau et al., 2016). The FDA has released guidelines for CGT products, regulated under the Code of Federal Regulations (CFR) 210, 211 that outline release testing. The guidance released by the FDA includes: demonstration of biological activity (potency); quantitative data; pre-defined acceptance and/or rejection criteria; employment of appropriate standards, controls, and reference materials; documentation of accuracy, sensitivity, specificity, and reproducibility of test methods; ingredient strength and identity; dating periods; and labeling requirements (Food and Drug Administration, 2011a; Galipeau et al., 2016).

Similarly, in Europe, clinical MSCs are considered an advanced therapy medicinal product (ATMP) in accordance with the European Medicines Agency (EMA) regulation 1394/2007 of the European commission (EC) (European Commission, 2007; Ancans, 2012; Rojewski et al., 2019). Under the ATMP, the identity and impurities of the MSCs must be described using the ISCT minimal criteria or a modification to the criteria (Horwitz et al., 2005; Dominici et al., 2006; European Commission, 2007; Wuchter et al., 2015; Rojewski et al., 2019). In addition, release criteria, which vary by type of clinical trial and requirements from other national competent authorities, are also governed under the ATMP and include contamination screening (microbial, endotoxin, and mycoplasma), viability, clonogenicity, identity, purity, and functional tests (European Commission, 2007; Ancans, 2012; Rojewski et al., 2019). Europe's regulatory approval process for cell therapy products is reviewed more thoroughly here (Ancans, 2012; Blasimme and Rial-Sebbag, 2013). Although, the ISCT made a point to clarify that their 2006 proposed guidelines should not be confused with final product release criteria, the ATMP regulations, along with the literature and FDA regulation submissions point to the fact that they may be seen as synonymous by some (Mendicino et al., 2014).

Although the FDA has released recommendations for developing tests to measure potency of the MSC product, the FDA does not provide recommendations regarding which specific assay should be used. Currently, each IND application is reviewed based on individual product attributes and is not compared to other MSC products (Galipeau et al., 2016; Galipeau and Senséb, 2018). Due to the biological nature and limited amount of the MSC product, hurdles exist that make development of assays and standardization difficult. Galipeau and Senséb (2018) review these challenges thoroughly and they list a number of problems such as variability of raw materials, limited product for testing, absence of appropriate standards, and *in vivo* fate of the product. For “biologics” (i.e., biologically-derived therapeutics) such as MSC-based therapeutics to be successfully manufactured at large scale, they must meet four criteria: (1) a stable and well-defined cell line; (2) a good manufacturing practice (GMP)-grade supply chain with a process control plan that has set variability values that produce a product with the desired therapeutic effect; (3) a standardized procedure that allows for process changes while maintaining product consistency; and (4) integrated redundancy and flexibility to allow for adaptation without sacrificing product consistency (Melsheimer et al., 2018). Even with these criteria met, biologics are still produced from living organisms and this variability causes product changes (e.g., quality, behavior, safety) that in turn affect the clinical use (Melsheimer et al., 2018).

An analysis of FDA IND applications by Mendicino et al. (2014) revealed variability in MSC tissue sources, manufacturing methods, and MSC characterization. Interestingly, it was noted that only 7 of the 9 ISCT-recommended MSC markers were ranked in the top 20 markers used by applicants to characterize human MSCs (Mendicino et al., 2014). In addition, they discovered that applications were submitted with MSC-characterization markers reported well below the 95% proposed by the ISCT, e.g., submissions with CD105 reported at only ~80%, although it is unclear whether this impacts MSC function or not (Mendicino et al., 2014). This data brings the ISCT guidelines into question. If the end goal is clinical use as an FDA-approved therapeutic, yet the FDA does not require the proposed criteria, and they are not consistently demonstrated by applicants, what purpose are they serving related to that goal? If applicants are struggling to meet these guidelines, how well are the guidelines serving the human MSC product? Further, how can it be expected that nonhuman MSCs will adhere to these standards? To combat MSC product inconsistencies and ensure successful clinical translation, variability in the process and product must be realized, described, and managed.

Additionally, as noted in a review from the FDA, MSC manufacturing reflects a broadening of MSC characterization release criteria that are associated with phased clinical testing (Mendicino et al., 2014). This is the opposite of what the FDA expects and is a double-edged sword—allowing cells which fail to meet MSC criteria in the released MSC product may have secondary consequences of reduced potency and increased lot-to-lot variation. It should be noted that although MSC characterization is not required by the FDA, generating a consensus MSC definition would benefit all MSC shareholders as it would enable comparison across studies and enable therapeutic use by producing more consistent effect sizes (Mendicino et al., 2014).

### MSC-Based Products Also Suffer From Lack of Standardization

MSCs being a product-by-process has implications that challenge the field, and it is a barrier to the idea that an MSC is a defined cell type. First, it implies that a process is necessary to generate or enrich cells of interest. Note that a similar notion is applied to pluripotent stem cells (PSCs), where the cells of interest are unnatural artifacts of the culture process and the culture conditions required to maintain them as immortal cells are known. In contrast, MSCs are mortal cells since the culture conditions needed to render MSCs as immortal cells are unknown. The product-by-process, together with the mortality of MSCs, implies that different MSC products are obtained at different times. Further, measures may reflect processes, and thus parse rather than unify.

The product-by-process assumption implies that prospective identification of MSCs is irrelevant since the product requires processing to be revealed. It also implies that different products are produced by altering the process. For example, “priming” MSCs by exposure to inflammatory cytokines can cause significant changes to MSCs such as inducing expression of MHC II (Romieu-Mourez et al., 2007; Tang et al., 2008). Moreover, the product-by-process focuses on *in vitro* and not the *in vivo* functionality of MSCs, and this is a key shortcoming to clinical translation.

If we embrace the product-by-process notion for MSCs, like we do PSCs, we can perhaps refocus efforts on what we can control and measure. For example, of the methods used to define MSCs, flow cytometry is the best method of cellular-level measurement that lends itself to metrology, i.e., a reference measurement system with traceability to the SI or other internationally agreed-upon units. In contrast, tri-lineage differentiation assays cannot be considered metrology as they lack defined measurands and reference materials. Therefore, we suggest that the MSC field develop and require measurable differentiation assays for publication.

It was once believed that the primary mechanisms of action for MSCs was contact-dependent signaling and engraftment into tissues, based on their potential for differentiation (Ankrum et al., 2014). In the past few years, it has become more widely accepted that MSCs' primary mechanism of action is through a paracrine effect. Through the paracrine effect, MSCs can secrete biologically active molecules, such as cytokines, chemokines, growth factors, extracellular matrix, and extracellular vesicles (EVs) (Liang et al., 2014). These molecules act therapeutically to stimulate tissue regeneration and angiogenesis as well as to modify inflammation, apoptosis, and fibrosis (Chen et al., 2009; Meirelles Lda et al., 2009; Ankrum and Karp, 2010; Linero and Chaparro, 2014). Due to their regenerative potential, EVs derived from MSCs (MSC-EVs) have become a target for therapeutic use. Preclinical data indicates that MSC-EVs may possess therapeutic behaviors similar to their parent cell of origin but with the additional benefit of using a cell-free product (Tögel et al., 2007; Yeo et al., 2013; Park et al., 2019). Although promising, the issue at hand is that without a consensus on the guidelines for characterizing an MSC, how can we logically move forward with MSC-based products? EVs isolated from conditioned media come with their own unique inconsistencies that can be due to parent cell of origin, the health of the cell donor, isolation and separation method, and storage condition (Li et al., 2019; Ludwig et al., 2019). Taken together with MSCs, the inconsistencies between the two products can only multiply when MSCs are used to manufacture EVs. Establishing guidelines for MSCs would further benefit EV research by allowing scientists to focus efforts on EVs rather than attempting to parse out inconsistencies from both sources.

### Tissue Source Differences

MSC-like cells have been found in many tissues but due to the fact that MSCs were first described in the bone marrow (BM), BM-MSCs have dominated the field and are the focus for the defining criteria. BM harvest is a painful and invasive procedure. BM-MSCs isolated from elderly donors have been shown to be less “stemmy,” and difficult, or sometimes impossible, to expand since they rapidly senesce (Pittenger et al., 1999; Stolzing et al., 2008). Here, “stemmy” is referring to cells within the MSC population with stem cell-like properties. Other adult tissue-derived MSCs such as adipose tissue (AT); dental pulp; muscle; and extra-embryonic tissues, such as the umbilical cord stroma, umbilical cord blood, and placenta, are also rich sources of MSCs (Wright et al., 2020). Some of these tissues, such as AT and extra-embryonic tissues, can be harvested rather easily secondary to routine or elective procedures. Furthermore, extra-embryonic tissues represent a painlessly-collected, virtually inexhaustible resource for MSC isolations. Consequently, they may represent an ideal source for MSCs because they are easily and painlessly obtained from donors of a consistent young age, hence minimizing the potential effects of aging or prior health conditions on the MSC pool.

Research groups may have a strong preference regarding which MSC tissue source they study and strong beliefs lead to claims of perceived superiority of a particular tissue source. Although there is consensus that MSCs derived from various tissues are not identical, the differences regarding characterization, and other behaviors, are often overlooked or perhaps exaggerated. The strongest evidence for this fact comes from the joint statement put out from the International Federation for Adipose Therapeutics (IFATS) and the ISCT in 2013 establishing an amended set of minimal guidelines for characterization of the uncultured stromal vascular fraction (SVF) and cultured stromal cells both derived from adipose tissue (Bourin et al., 2013). Importantly, these guidelines acknowledge that SVF can be CD34+ and adds CD44 (positive) and CD31 (negative) to the panel for cultured adipose-derived MSCs (Bourin et al., 2013). Interestingly, tissue-specific guidelines do not exist for other sources.

The literature highlighting tissue-specific MSC differences is vast but can often be conflicting and difficult to interpret. For example, umbilical cord-derived (UC-MSCs) and adipose-derived (AT) MSCs have been shown to have a higher proliferative capacity when compared to BM-MSCs (Kern et al., 2006; Lu et al., 2006; Baksh et al., 2007; Chen et al., 2009; Wu et al., 2009; Hass et al., 2011; Yu et al., 2018). Lu et al. (2006) reported a constant population doubling time (PDT) for human UC-MSCs passage 1−10 of ~24 h compared to a PDT of ~40 h for BM-MSCs, which increased significantly after passage 6. Peng et al. (2008) not only reported different PDTs of rat AT-MSCs compared to BM (45.2 h compared to 61.2 h, respectively) but also noted that BM-MSCs are morphologically larger than AT-MSCs. In regards to differentiation potential, BM-MSCs have been shown to have increased osteogenic potential and decreased adipogenic potential compared to AT-MSCs (Danisovic et al., 2009; Xu et al., 2017). Chen et al. (2009) demonstrated that although human BM- and UC-MSCs have similar adipogenic, chondrogenic, and osteogenic potential, UC-MSCs have a higher endothelial differentiation potential making them ideal for neovascularization of engineered tissues. Work reported from gene expression pathway analysis suggests that MSCs derived from human UC and amniotic membrane may possess an increased immunomodulatory capacity compared to BM-MSCs, while BM-MSCs have a higher potential for neuronal differentiation and development (Wegmeyer et al., 2013). Interestingly, in human placenta-, UC-, and amniotic membrane-derived MSCs, CD105, and CD29 expression was found to be negatively correlated to maternal age (Alrefaei et al., 2015, 2019). In equines, gene expression data found significant differences in CD44, CD90, CD29, and CD34 between BM and AT-MSCs (Ranera et al., 2011).

### Species Differences

The ISCT's MSC definitions were based upon human BM-MSCs yet a large portion of MSC preclinical work is done in other species. Similar to pluripotent stem cells (PSCs), human MSCs are likely to have different characteristics than MSCs derived from other animals. To further complicate the matter, human MSCs also share some defining characteristics with animal MSCs, as shown in the case of human PSCs compared to rat and mouse PSCs (Schnerch et al., 2010). These similarities and differences between MSCs across species should be embraced to gain consensus and uniformity in the field (Tropel et al., 2004; Hu et al., 2018; Uder et al., 2018). Additionally, availability and reliability of many antibodies against key surface markers are disparate across species, making it difficult to find reliable information for MSC characterization (Wright et al., 2020). Hence, it can be difficult to determine whether characterization differences are true differences or an artifact of antibody selection/performance.

Further, the tri-lineage differentiation potential of MSCs derived from nonhuman species is similar but not identical (Chamberlain et al., 2007; Uder et al., 2018). Scuteri et al. (2014) showed that BM-derived rat MSCs vary in their differentiation potential compared to BM-derived human MSCs in standard culture conditions. In terms of osteogenic and chondrogenic differentiation, the time required for differentiation was different between rat and human MSCs, while in adipogenic differentiation, human MSCs had a greater capacity than rat MSCs (Scuteri et al., 2014). In the canine MSC literature, it has been proposed that differentiation to two lineages is sufficient for characterization rather than three (Chamberlain et al., 2007; Neupane et al., 2008; Djouad et al., 2009; Vieira et al., 2010; Wood et al., 2012). In our review of 46 canine MSC papers, 22 (48%) demonstrated differentiation to three lineages. Of the remaining papers, 11 (24%) demonstrated differentiation to 2 of the lineages, and 10 (22%) papers did not address differentiation of the MSCs in any capacity (Wright et al., 2020). Of those, the most common lineage not shown, or not successful, was chondrogenic, which can be difficult (Zhang et al., 2015).

One similarity that all species seem to share is that differentiation potential decreases as cumulative population doublings increase. This attribute appears to be consistent among all lineages, species, and tissue sources (Requicha et al., 2012; Volk et al., 2012; Sasao et al., 2015; Marín-Llera and Chimal-Monroy, 2018). This evidence indicates that a true property of MSCs perhaps is a loss of potency, or “stemness,” with time in culture. Despite this common feature, no priority has been placed on developing a standardized quantitative assay to measure differentiation or setting a standard number of cumulative population doublings at which differentiation potential should be assessed. In many cases, that information is not provided in MSC literature.

Mouse BM-derived MSCs have been shown to vary notably from human MSCs in their surface marker expression, specifically in the instance of CD34 (Chamberlain et al., 2007). Hu et al. (2018) demonstrated that BM-MSCs from C57BL/6 mice expressed high levels of CD34 but lacked CD90 as well as noted slight strain differences in surface marker expression. In our laboratory, canine MSCs derived from the UC require different culture conditions with regard to attachment factors, media formulation, and lifting agents compared to human UC-derived MSCs (Smith et al., 2017; Wright et al., 2020). Further, we have demonstrated that canine UC-MSCs express CD34 and CD90, albeit CD90 expression is not as high as human UC-MSCs (Wright et al., 2020). While others have also shown that canine MSCs express CD34, this finding raises concerns about the similarities of MSCs from different species (Kang et al., 2008; Ryu et al., 2009; Russell et al., 2016). AT-derived MSCs from rhesus monkeys and horses were shown to have related biological properties to human MSCs but differ in expression of surface markers and proliferation rates (Izadpanah et al., 2006; Ranera et al., 2011; Uder et al., 2018). AT-derived MSCs from rats and mice have also been shown to exhibit similar yet different surface marker expression compared to human AT-MSCs (Taha and Hedayati, 2010; Jeong et al., 2014; Uder et al., 2018).

**Figure 1:**
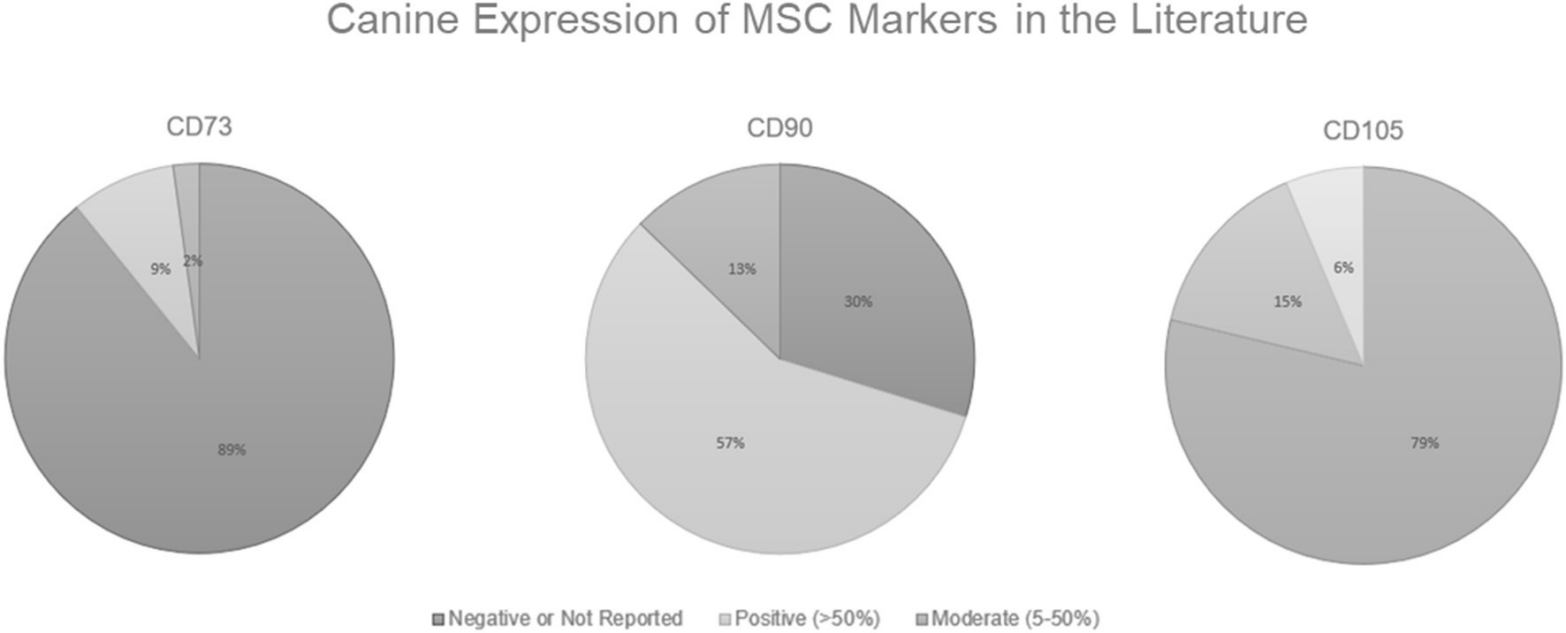
Canine Expression of MSC Markers in the Literature. Positive expression is defined here as >50% surface marker expression, moderate is defined as ≥5%— <50%, and anything <5% is considered to be a negative result. Data derived from Wright et al. (2020).

As shown in Figure 1, in the canine MSC literature, there is a problem with demonstrating surface marker expression of all 3 classic MSC markers designated by the ISCT (CD73, CD90, and CD105). Some researchers believe that positive expression of CD44 and CD90 along with the negative expression of CD34, CD45, CD80, CD86, or MHC II is sufficient to characterize canine MSCs (Chamberlain et al., 2007; Neupane et al., 2008; Djouad et al., 2009; Vieira et al., 2010; Wood et al., 2012). Of the 46 papers reviewed, 41 (89%) either had negative results or did not report results for CD73, while only 4 (9%) had positive results (generously defined as >50% surface marker expression), and 1 (2%) had moderate expression (as defined as ≥5%— <50%). Note here the discrepancy in “positive” expression. The ISCT definition dictates that the MSCs should have ≥95% surface marker expression to be deemed positive yet instances exist of researchers stating positive results in populations with <50% expression. While CD90 expression was most consistently reported, only 27 (57%) of papers reviewed had positive expression. For CD105 expression, 37 (79%) of the papers reviewed had negative or unreported results. Bearden et al. (2017) reported that not only was CD105 expression more variable in canine MSCs than seen in humans, but it was also variable among canine MSC tissue sources. In the flow cytometric analysis of canine MSCs isolated from adipose, bone marrow, and synovium at the same passage, CD105 expression in MSCs derived from adipose (~60%) and synovium (~46%) was significantly higher than from bone marrow (~17%) (Bearden et al., 2017).

Although some researchers report that MSCs are positive for a certain surface marker, what designates a positive expression is not clear and can be seen as subjective. The ISCT standards state that MSCs should be ≥95% expression for humans and other species are often held to this same standard (Dominici et al., 2006). We, and others, have only been able to demonstrate positive expression by approximately half, or even less, of the population (Radcliffe et al., 2010; Hermida-Gómez et al., 2011; Kisiel et al., 2012; Takemitsu et al., 2012; Screven et al., 2014; Escalhão et al., 2017; Kovac et al., 2017; Liu et al., 2018; Long et al., 2018; Wright et al., 2020). In the canine literature, this seems to be an issue with CD90 in particular (Kisiel et al., 2012; Takemitsu et al., 2012; Screven et al., 2014; Liu et al., 2018; Long et al., 2018; Wright et al., 2020). Further, in earlier published work, we demonstrated that there was no difference in expression between an antibody raised specifically to canine for CD90 and a human antibody with canine cross-reactivity (Wright et al., 2020). Either there is lower expression of CD90 in canine MSCs or there are issues with antibody specificity.

In a review of MSCs derived from other species, all species noted some difficulties exhibiting expression of the 3 classic MSC markers. In the equine literature, CD73 and CD105 are most often unreported or negative (de Mattos Carvalho et al., 2009; Radcliffe et al., 2010; Ranera et al., 2011; Maia et al., 2013; Barberini et al., 2014; Alipour et al., 2015; Zahedi et al., 2017; Gale et al., 2019; Kamm et al., 2019; Lepage et al., 2019). In mouse literature, there are several examples of researchers being able to demonstrate one marker and not the other two, but no clear pattern as to which marker is shown to have positive expression (Meirelles Lda and Nardi, 2003; Anderson et al., 2013; Hosseinzadeh Shirzeily et al., 2013; Deng et al., 2014; Li and Niyibizi, 2016; Ahmed et al., 2017). In rat literature, there are also several examples of researchers being able to demonstrate one marker and not the other two, with all of the examples including CD105 as one of the two surface markers missing or negatively expressed (Rui et al., 2010; Meric et al., 2013; Sobh, 2014; Sarvandi et al., 2015; Suto et al., 2017). Porcine (Ock et al., 2010; Brückner et al., 2013; Lee et al., 2015; Pérez-Serrano et al., 2017; Wiater et al., 2018), ovine (Fadel et al., 2011; Czernik et al., 2013; Ji et al., 2016), rabbit (Lee et al., 2013; Xia et al., 2013; Kovac et al., 2017; Xiao et al., 2018), bovine (Corradetti et al., 2013; Gao et al., 2014; de Moraes et al., 2016; Yue et al., 2018), buffalo (Ghosh et al., 2015), and chickens (Bai et al., 2013) also demonstrate negative or missing classic MSC surface marker expression with no clear pattern or rationale. Interestingly, Kamm et al. (2019) noted significantly higher CD90 cell surface expression in MSCs derived from universal blood donor Standardbred equines compared to non-blood donor Standardbreds.

There is no way to know for certain if the negative results are true negatives, alluding to the fact that surface marker expression of MSCs varies by species, or if the antibody availability is limited for other species causing false negatives. There is evidence for both claims leading us to believe that it is a combination of the two. Researchers have demonstrated that these markers are present at the mRNA level, even if the protein expression is negative or not strongly positive (Requicha et al., 2012; Crain et al., 2019; Wright et al., 2020). Although not equal to showing surface marker protein expression, the fact that researchers feel compelled to demonstrate classic MSC markers at the mRNA level, yet cannot produce ISCT-standard flow cytometric data, brings the surface marker panel for MSC characterization into question. By holding MSCs from nonhuman species accountable for human characterization criteria, are we excluding valuable data from the field? Instead, we should be working toward a new consensus that makes accommodations for non-human MSCs.

### MSC Heterogeneity

When considered jointly, the definition of an MSC and the ISCT minimal defining criteria contradict one another. On one hand, there is the definition of MSCs—a heterogeneous population that includes stem, progenitor, and differentiated cells. On the other hand, there are the guidelines for demonstrating that these cells are indeed MSCs, which includes plastic-adherence, tri-lineage differentiation, and a panel of positive and negative surface markers in which the positive should be expressed in ≥95% of the population (Dominici et al., 2006). Where did 95% come from? It may be unrealistic to assume that a heterogeneous population of cells, derived by different methods, from different tissues and species, may be able to demonstrate such high expression of a single marker, let alone an entire panel. Perhaps in the journey to reach a consensus on what an MSC is, the actual intent has been lost.

In addition, the definition of an MSC includes those cells from all tissues, yet the guidelines were established for human BM-MSCs. Researchers have been liberal with applying these guidelines to MSCs from many tissue sources and species. This act alone implies that MSCs isolated from different tissues and species are phenotypically and functionally similar. MSCs are not uniform and to insist that they are is unnecessarily forcing a round peg into a square hole. There is considerable evidence pointing to differences in MSCs derived from different culture conditions, different tissue sources, different aged donors, and different species. These differences are exhibited in MSC surface marker expression, their culture requirements, their longevity in culture, their transcriptome, their response to stimulation, and their growth rate. Taken together, this alludes to the fact that a simple definition might not properly serve all MSCs.

### Purity vs. Potency

The issue remains that the characterization guidelines are nonspecific and, as discussed above, MSCs are a heterogenous population of cells with different gene expression profiles, differentiation and proliferation potential, and phenotype, which are all influenced by donor age, tissue source, species of origin, isolation procedure, and culture conditions (de Wolf and van de Bovenkamp, 2017). It is still unclear whether surface marker characterization, which is meant to assess the purity of the population, is correlated to functional activity, or potency of the MSCs. To combat this, most researchers use a functional assay to demonstrate potency of the cells. The assay should relate to the intended therapeutic MOA, but assays are left to the discretion of the researcher. At this time, it is still unclear whether *in vitro* functional assays correlate to *in vivo* activity, and that assumption is a major flaw with potency measures.

### Need for an Expanded Surface Marker Characterization Panel

Even with the species variations considered, there are surface markers that are more uniformly expressed on MSCs of all species that are often included in flow cytometric panels (even in commercially available kits), and are thought of as “standard” MSC markers– yet they are not included in the ISCT characterization guidelines. Expression of CD44 and CD29 should be considered as logical additions to the MSC surface marker panel and adding them may give researchers working with nonhuman species additional options for MSC characterization.

CD44 is a hyaluronic acid receptor and a critical adhesion molecule. CD44 has been found to be highly expressed on MSCs derived from human (Hu et al., 2003; Le Blanc et al., 2003; Wexler et al., 2003; Brooke et al., 2008; Park and Patel, 2010; Lee et al., 2011; Aldridge et al., 2012; Liu et al., 2012; Guan et al., 2014; Qu et al., 2014; Secunda et al., 2015; Katsiani et al., 2016; Van Pham et al., 2016; Smith et al., 2017; Togarrati et al., 2018; Kaviani et al., 2019), canine (Filioli Uranio et al., 2011; Choi et al., 2013; Screven et al., 2014; Ivanovska et al., 2017; Zhang et al., 2018; Wright et al., 2020), equine (de Mattos Carvalho et al., 2009; Radcliffe et al., 2010; Maia et al., 2013; Barberini et al., 2014; Alipour et al., 2015; Sasao et al., 2015; Zahedi et al., 2017; Kamm et al., 2019; Lepage et al., 2019), mouse (Meirelles Lda and Nardi, 2003; Valorani et al., 2010; Deng et al., 2014; Fujita et al., 2015; Ahmed et al., 2017; Naik et al., 2017), rat (Rui et al., 2010; Yang et al., 2010; Meric et al., 2013; Sobh, 2014; Sarvandi et al., 2015; Li et al., 2020), rabbit (Lee et al., 2013; Xia et al., 2013; Kovac et al., 2017; Xiao et al., 2018), buffalo (Ghosh et al., 2015; Deng et al., 2018), bovine (Corradetti et al., 2013; Gao et al., 2014; de Moraes et al., 2016; Yue et al., 2018), porcine (Brückner et al., 2013; Lee et al., 2015; Pérez-Serrano et al., 2017; Wiater et al., 2018), ovine (Fadel et al., 2011; Czernik et al., 2013; Chen et al., 2018), and chickens (Bai et al., 2013). CD44 expression is often associated with cell proliferation and migration (Yang et al., 2010; Azghadi et al., 2016; Ouhtit et al., 2020). It has been reported that CD44 expression in MSCs, both human and mice, is a product of *in vitro* culture as freshly isolated MSCs do not express CD44 until after cultured (Qian et al., 2012). On the contrary, some have demonstrated that CD44+ primary isolates are present (Hachisuka et al., 2007; Radcliffe et al., 2010; Fujita et al., 2015; Marín-Llera and Chimal-Monroy, 2018). Many researchers have documented increased CD44 expression on MSCs of multiple species with time in culture (Park and Patel, 2010; Radcliffe et al., 2010; Qian et al., 2012; Marín-Llera and Chimal-Monroy, 2018) with only minimal evidence of CD44 expression decreasing as time in culture increases (Sasao et al., 2015). Since flow cytometry assesses cell surface markers, the dissociation of MSCs using trypsin is also problematic due to cleavage or disruption of antigens. For example, trypsin dissociation significantly reduces CD44 expression, as well as other MSC surface markers, on human MSCs compared to other dissociation agents such as TrypLE (Tsuji et al., 2017). Further, CD44 expression may also affect the chondrogenic differentiation of human MSCs via the Smad 2/3 and ERK ½ signaling pathway (Xu et al., 2020). In UC blood-derived MSCs, Kwon et al. (2019) demonstrated that CD44 has an immunoregulatory role as evidenced by the induction of macrophage polarization via CD44 expression by the proteoglycan, decorin.

CD29, integrin beta-1, is a cell surface receptor that is involved in cell adhesion. CD29 has been found to be “highly” expressed (≥95%) on MSCs derived from human (Hu et al., 2003; Le Blanc et al., 2003; Wexler et al., 2003; Brooke et al., 2008; Pruszak et al., 2009; Park and Patel, 2010; Aldridge et al., 2012; Al-Nbaheen et al., 2013; Guan et al., 2014; Yang et al., 2014; Alrefaei et al., 2015; Katsiani et al., 2016; Van Pham et al., 2016; Togarrati et al., 2018; Kaviani et al., 2019), rat (Wu et al., 2009; Walker et al., 2010; Song et al., 2014; Davies et al., 2015; Suto et al., 2017), equine (Ranera et al., 2011; Alipour et al., 2015; Esteves et al., 2017; Zahedi et al., 2017; Gale et al., 2019; Lepage et al., 2019), canine (Filioli Uranio et al., 2011; Choi et al., 2013; Ivanovska et al., 2017), mouse (Meirelles Lda and Nardi, 2003; Ahmed et al., 2017), porcine (Ock et al., 2010; Lee et al., 2015; Wiater et al., 2018), buffalo (Deng et al., 2018), rabbit (Lee et al., 2013; Kovac et al., 2017), bovine (Corradetti et al., 2013; de Moraes et al., 2016), and chickens (Bai et al., 2013). Evidence suggests that CD29 expression may be involved with MSC migration along with CD73 (Ode et al., 2011). CD29 and CD105 expression has been found to be negatively correlated with maternal age on human placenta- and UC-derived MSCs and was proposed as a marker for quality control (Alrefaei et al., 2015, 2019). Both CD29 and CD44 expression were found to be involved with MSC adhesion, migration, and engraftment in the diseased liver (Aldridge et al., 2012).

A total of 72% of canine papers demonstrated either a single alternative MSC marker (CD29 or CD44) or both, which is more consistent than any of the classic MSC markers (Figure 1). This remains true with all other species examined here. All species noted here were able to demonstrate expression of either CD29, CD44, or both as a positive surface marker and at levels >50% of the population. Because of this, we believe that both CD29 and CD44 are logical additions to the MSC markers for all species, due to their demonstrated high expression levels and inclusion within all species. Although both CD29 and CD44 are expressed on epithelial cells, epithelial cells do not express the classic MSC markers CD105, CD90, and CD73, hence CD31 could be added as a negative marker for MSC characterization (Seeberger et al., 2009; Togarrati et al., 2018). The addition of CD44 and CD31 has already been done in the IFATS guidelines for cultured adipose-derived MSCs (Bourin et al., 2013).

Other markers, such as Stro-1, CD271, CD362, and ABCB5, are also considered as MSC markers by some researchers and even used for MSC flow sorting (Ning et al., 2011; Álvarez-Viejo et al., 2015; Ballikaya et al., 2020; Gonzalez et al., 2020). However, in our review we did not find these antibodies to be as available for other species or as well-demonstrated in the literature as CD29 and CD44. For those reasons we suggest CD29 and CD44 as the next logical additions to the MSC panel. Perhaps attempting to make generalized criteria to define MSCs from any tissue source, any species, and any culture conditions is too simplistic. Rather, an updated species- and tissue-specific set of criteria could better serve the field of MSC research given that they are specific and reproducible (Keating, 2012). Further, MSCs may represent different products, and treating them as homogeneous may impede new work in the field.

### Metrology Standards

It is recognized that the MSC definition casts a “wide net” as it does not rely upon a single cell surface marker or activity assay that can prospectively identify the stemmy population within the mixed population. In lieu of a single surface marker, a surface marker analysis panel, consisting of both positive and negative markers, is one key element to defining MSCs. There is a vast amount of literature that addresses the flow cytometric analysis of MSCs, and it is quite challenging to compare the results between laboratories (Uder et al., 2018).

In response to this issue, some experts have proposed that MSC lines be generated and highly characterized to serve as “gold standard” lines for calibration (Viswanathan et al., 2014; Tanavde et al., 2015). Others have suggested the use of dedicated laboratories to serve as characterization centers for MSCs to enable standardized characterization in the field, as has been done with certain diagnostic tests. We find that both of these proposals come with their own advantages and disadvantages. A third, and perhaps more realistic consideration might be to forgo the simplified definition of an MSC in favor of guidelines that are specific to the species and tissue used to generate the MSCs. Generating a consensus sponsored by the ISCT around authentication methods and materials, e.g., specific monoclonal antibody clones, protocols, and criteria regarding positive and negative staining, as well as a consistent presentation of results, would enable reproducibility and comparison across laboratories.

Since the National Institutes of Health (NIH) and National Science Foundation (SF) require authentication of biological reagents, we suggest that cellular metrology standards be set, just as they have been for other biologicals such as microbiology strains, bacteria, and cancer cell lines. Standards set by the community should provide guidance for publication, reproducibility requirements, and authentication standards. It is our belief that the ISCT should establish MSC metrology guidelines by species and tissue source; generate a consensus-gathering list of available and acceptable resources for characterization by species and tissue source; and enumerate guidelines that dictate the minimal information required for published MSC studies that includes characterization, methodology, and reproducibility requirements.

## Research Driven by Commercial Applicability

Despite the nuances, a shared trait among all MSCs is that they possess unique and tissue-specific differences in immunomodulatory properties and regenerative potential. To simply take advantage of these unique features and push MSCs to market for therapeutic use is not feasible. Questions remain concerning the mechanism of action, how *in vitro* testing correlates to *in vivo* activity, the number of cells in a dose, the route of administration, and how all of this relates to the therapeutic effects for the various indications (Mendicino et al., 2014). To properly address these concerns, more research funding is required.

In the United States, R&D is primarily funded through the federal government, state governments, businesses, academia, and nonprofit organizations. From historical data dating back to 1953, businesses and the federal government combined have accounted for over 90% of the R&D expenditures (Sargent, 2020). While the federal government suffered 7 consecutive years of declines in funding (2009-2016), businesses have increased funding since 1953 (Sargent, 2020). In the most recent data for the fiscal year 2018 released this year, the federal government spent $127.3 billion on R&D while businesses spent $404.2 billion and state governments, academia, and nonprofit organizations spent a combined $48.5 billion (Sargent, 2020). Although it cannot be parsed out exactly where these funds were distributed, the point can be made that businesses are spending 2-4x more money on R&D than the US government. In a search of sponsored clinical trials in the United States (clinicaltrials.gov, search MSC, all trials, US, 7/29/20), other sponsors (individuals, universities, and organizations) accounted for almost half of the 1,195 total registered clinical trials (578), while industries sponsored 368, and NIH and other federal agencies accounted for 279, the smallest pool.

Research supported by federally-funded grants is fundamentally different from industry-sponsored research. While both are critical to moving science forward, federally-funded research addresses questions aiming to fill a void of knowledge. Industry-sponsored research is more focused on topics with a clear commercial application and an established large market share (Fabbri et al., 2018). For example, work examining biomedical research funding in the United States from the early 2000s found that industries were more likely to sponsor research centered around diseases projected to afflict areas of higher income as opposed to NIH funding targeting diseases with a global burden (Dorsey et al., 2009; Fabbri et al., 2018). MSCs represent an attractive research topic because they have applicability for numerous indications with widespread prevalence, an established market share, and the potential to outperform many standard of care therapies. Research focused on the big picture, i.e., commercial use of MSCs, could attract more industries looking to enter the MSC market, thus leading to increased research funds from industry sponsors. Here, we will compare the market of allogeneic and autologous MSC therapy. We should note that there are many other factors to take into consideration such as shipping logistics, cryopreservation, culture conditions, and manipulations to alter therapeutic effect (e.g., priming) that are not addressed here.

### Allogeneic MSC Therapy Represents a Viable Business Model

MSCs can be used therapeutically in either an autologous or allogeneic manner and both have their own unique set of benefits and limitations. Autologous MSC therapies are considered a lower risk than allogeneic therapies for humans with intact immune systems. The two types of therapies are not synonymous and the results cannot be compared across clinical trials. Further, within allogeneic and autologous therapies, other factors such as preparative regimen, administration method, disease models, the dosage of MSCs administered, and the use of either culture-expanded or cryopreserved cells should also be carefully considered before comparing results, as they possibly impact therapeutic effectiveness of MSCs and the cells' ability to meet primary endpoints.

Autologous MSCs are a form of personalized medicine and are of less risk immunologically since they are one's cells. However, autologous MSCs typically require *in vitro* culture-expansion to produce enough cells to constitute a therapeutic dose. Hence, they are limited to situations in which time is not a critical factor and collection is feasible. Turnaround times from harvest to patient administration can vary widely due to the variable proliferation rates among patients and the number of cells required for a therapeutic dose. Further, MSCs have been shown to be less efficacious when harvested from elderly donors, thus limiting the potential patient pool (Lepperdinger, 2011; Alt et al., 2012). The high cost of autologous MSC therapy coupled with the lack of insurance coverage makes it unattainable for the majority of possible recipients. Despite causing heavy criticism and providing risky services that claim to provide unproven results, unregulated “stem” cell clinics around the world demonstrate that the market demand for cell therapy exists. In fact, the global market demand for MSCs is expected to reach $7.5 billion USD by 2022, with the US expected to have the largest market share (34.3%) despite the fact that the US has yet to grant approval to an MSC product (Pereira Chilima et al., 2018). It should be noted that unregulated stem cell clinics operate using a “minimally-manipulated” product or a homologous lipoaspirate [21 CFR 1271.10(a)(1) and 21 CFR 1271.10(a)(2), respectively]. It is unclear whether or not this will continue to be an exempt product in the future. It should be noted that MSCs are not considered minimally manipulated since they require *in vitro* expansion and thus are not exempt.

**Figure 2:**
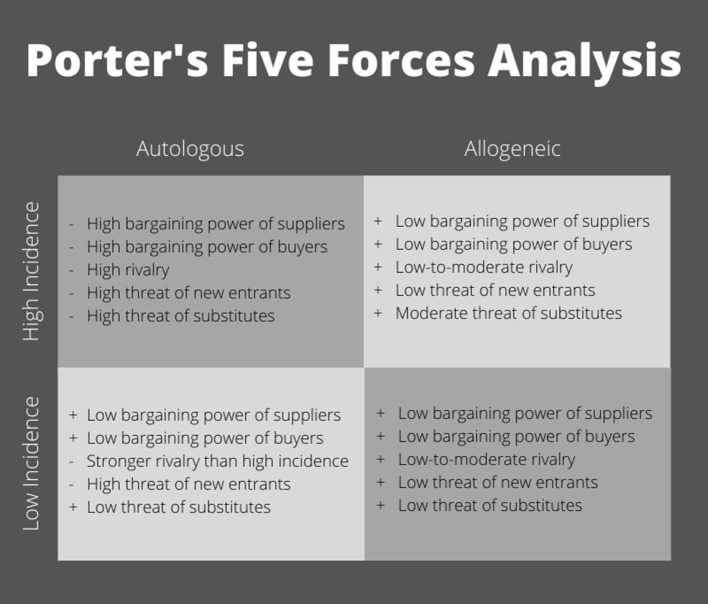
Porter's five forces analysis of competitive environment within MSC clinical use.

Industry sponsors have funded the majority of advanced phase clinical trials (Ankrum and Karp, 2010; Galipeau and Senséb, 2018). Without industry support, getting MSC products approved for use is cost prohibitive. To gain industry backing, a clear path to profitability must be established in a manufacturing market that is driven by margins. To explore potential markets, let us apply a standard business model used to analyze industry profitability (Figure 2). Michael Porter's “five forces” approach to industry analysis examines the broader industry structure to determine the overall attractiveness of an industry for investment (Porter, 2008). In addition to interfirm rivalry, profit potential is determined by the threat of new entrants, the availability of attractive substitutes, and the power of suppliers and buyers, respectively.

The most logical pathway to commercialization is to target a sizable indication with a high incidence rate (Figure 2). Applying Porter's five forces model, autologous cell therapy does not appear to have the ability to produce an adequate profit pool. The industry is fully reliant on donors' willingness and suitability to provide the key input (autologous cells) as well as their desire and ability to pay (e.g., high buyer and supplier power). Due to the nature of the manufacturing process for autologous cells, production processes are not scalable. Large batch manufacturing is not cost effective; as a result, production remains dominated by small, local laboratories. Without economies of scale to serve as an entry barrier, autologous MSC therapy has a high risk of new entrants, making for a highly competitive environment. Further, it is worth clarifying that there are two patent pathways: cell line and production/differentiation techniques. With autologous MSC therapy, cell lines, although more easily patentable and marketable, are moot and this leaves process patents. As evidenced in iPSC technology, process patents come with unique challenges such as a low number of approvals compared to applications (e.g., only 11% of applications approved by the European Patent Office with 89% waiting to be reviewed), differences in international intellectual property laws, and small patent portfolios distributed among several entities (Zachariades, 2013; Roberts et al., 2014). Particularly, patents are an issue in Europe where exemptions to patentability exist that may affect stem cell therapeutics, specifically the “use of human embryos for industrial or commercial purposes” (Zachariades, 2013). Additionally, patents can be seen as risky since the regulatory approval process to get cell therapies to market is quite long; patents may expire before the technology can be utilized commercially (Roberts et al., 2014). Because of this, many companies rely on trade secrets, which allow for processes to be improved and protected from common knowledge, but alleviate the concern of expired patents, making trade secrets a more viable alternative to intellectual property (Roberts et al., 2014). So, in the case of autologous cell therapy, you're left with a splintered landscape of patents and trade secrets where companies are forced to “brand” their technology to convince the market that they have any sort of strategic competitive advantage. This leaves a perfectly competitive market of a wide range of technological advances where it is difficult for brands to build brand recognition and demand a premium price compared to other competitors. Autologous MSC therapy also has a high risk of substitution. A majority of high incidence indications already have an existing standard of care produced using efficient manufacturing processes that have been refined over the years. To even be considered, autologous MSCs would have to demonstrate therapeutic benefits and safety beyond currently approved modalities to justify the higher cost. In this context, multiple, small firms would be forced to compete based on price alone (i.e., perfect competition), narrowing profit margins even further and making autologous cell therapy an unattractive industry to enter.

In contrast, allogeneic MSCs have the potential to be a readily-available product that can serve in instances of acute disorders where time to culture-expand cells is not feasible, or as an option for patients who are not able to serve as their own donor. Because allogeneic MSCs may be produced from a wider pool of “qualified” donors, producers have much greater control over their supply chain. Meanwhile, manufacturing processes for allogeneic cell therapies are more closely related to other noncellular pharmaceuticals and biologics. Based on these similarities, protocols for culture-expansion of cells in smaller batches could easily be scaled up using existing technologies and equipment. Particularly if automated, large-scale manufacturing of allogeneic cell therapies would spread the cost of goods, labor, and quality control across more samples, thus lowering the cost of production per sample, making this option ideal to treat large numbers of patients. Due to economies of scale, allogeneic MSCs would face a lower risk of new entrants and fewer overall competitors. As an off-the-shelf product, allogeneic MSCs must be licensed and approved for treatment by the FDA. The time and costs associated with regulatory licensing as well as the high costs of capital (e.g., equipment, facilities, and trained staff) needed to manufacture allogeneic MSCs at a large scale represent additional barriers to market entry. Allogeneic cell therapy has a substitution risk but due to the lower cost, it may be able to compete effectively with existing standard of care therapies, especially if it can demonstrate superior safety and efficacy. Marketing these cells under a brand name, utilizing the pharmaceutical industry's sophisticated marketing capabilities, could help allogeneic MSCs to build brand recognition, thus commanding a price premium. The ability to differentiate based on quality combined with cheaper costs of production would increase firms' power over “buyers,” who be more willing to pay a price premium for an approved therapy. From this standpoint, allogeneic MSCs represent a viable business venture.

An alternative “industry” to consider is an indication with a low incidence in which a standard of care may not exist or one with nonresponsive patients (Figure 2, bottom half). Autologous MSCs could be an option for treatment, if not time-constrained, due to the lack of available substitutes. Without the ability to scale, manufacturing costs would still be high, but buyers would be less price-sensitive and willing to pay a premium for a product with demonstrated efficacy, especially given the lack of a standard treatment option. By definition, an indication with a low incidence would have a small market size. Because of the low entry barriers, new laboratories could still join the industry, but the lack of growth potential would result in an increased level of rivalry. While this scenario is modestly improved compared to the high incidence quadrant, allogeneic MSCs again represent the more commercially viable option. With their broader pool of donors (suppliers), allogeneic MSCs can increase production to meet demand, thus benefiting from economies of scale. Due to the lack of substitutes and decreased price-sensitivity of buyers, firms could demand a premium price for a product with demonstrated efficacy, increasing profits. Again, with an allogeneic product, higher market entry barriers exist due to licensing and the costs of startup at scale. The ability to differentiate products decreases both the intensity of rivalry and the threat posed by new entrants. Although the overall market size is notably smaller, allogeneic MSCs still represent an attractive industry in terms of profitability.

Biologics have been successful on the market—over 250 products are available and they account for seven of the top 10 selling drugs globally—and several companies have already taken advantage of the allogeneic MSC model to produce clinical therapeutics (Melsheimer et al., 2018). There are well-established companies such as, JCR Pharmaceuticals [Japan], Mesoblast [Australia], and Osiris Therapeutics [United States], with new biotechnology companies opening worldwide regularly. Prochymal from Osiris Therapeutics was granted conditional licensing approval to treat children suffering from acute graft vs. host disease (GvHD) in Canada in 2012 (Galipeau and Senséb, 2018; Chisholm et al., 2019). It was revealed in 2016 that Prochymal had not been utilized because it could not get reimbursed (David Gagnon, 2016; Galipeau and Senséb, 2018). On the other hand, JCR Pharmaceuticals has had financial success with its product, TEMCELL®, which was approved for use in acute GvHD in 2015 (Galipeau and Senséb, 2018). From JCR Pharmaceuticals' financial reports, they have reported revenue of ¥86.6 billion (~817,400,000 USD) from fiscal years 2016–2019, with revenue increasing annually, and an operating income of ¥14.4 billion (~135,919,000 USD) (JCR Pharmaceuticals Co L, 2017, 2018, 2019, 2020). Collectively, these data indicate that allogeneic MSC therapy represents the clearest path to profitability. By focusing research efforts on this modality, industry-sponsored funding may increase.

## Conclusion

The cell therapy market is expected to grow to $61 billion by 2022 (Pereira Chilima et al., 2018). MSCs are an attractive cellular therapeutic product backed by promising preclinical data in animal models. There are currently ten MSC therapeutics with regulatory approval worldwide. Despite the positive preclinical data, in the US clinical trials have failed to meet efficacy endpoints, pointing to issues with translation from preclinical studies to clinical trials. Because of this, an FDA-approved MSC therapeutic product still does not exist. Unified under the common goal of widespread therapeutic use of MSCs, stakeholders should focus efforts on strengthening preclinical data so that it can be translated into safe and effective therapies, replicated among researchers, and compared across laboratories. To accomplish this, characterization guidelines should be updated to accommodate MSC populations from all tissue sources and species. Second, improved standardization that has both general characteristics and specific characteristics for each MSC population should be generated to decrease product variability. To accomplish this, research with commercial applicability should be prioritized to attract industry research funds. Without established consistency among MSCs, both MSCs and MSC-based products, such as EVs, will suffer from a lack of standardization, increasing the time to market as a licensed therapeutic.

## Author Contributions

AW and MW conceived and wrote the manuscript. MA-D contributed to the commercialization portion of the manuscript. MW generated financial support for the research. All authors approved the final version submitted for consideration.

## Conflict of Interest

The authors declare that the research was conducted in the absence of any commercial or financial relationships that could be construed as a potential conflict of interest.
